# Supplementary figures and images for: Cytotoxicity of Vibrio parahaemolyticus AHPND toxin on shrimp hemocytes, a newly identified target tissue, involves binding of toxin to aminopeptidase N1 receptor
Source: PLoS Pathog. 2021 Mar 26;17(3):e1009463. doi: 10.1371/journal.ppat.1009463 (PMC8041169; doi:10.1371/journal.ppat.1009463)

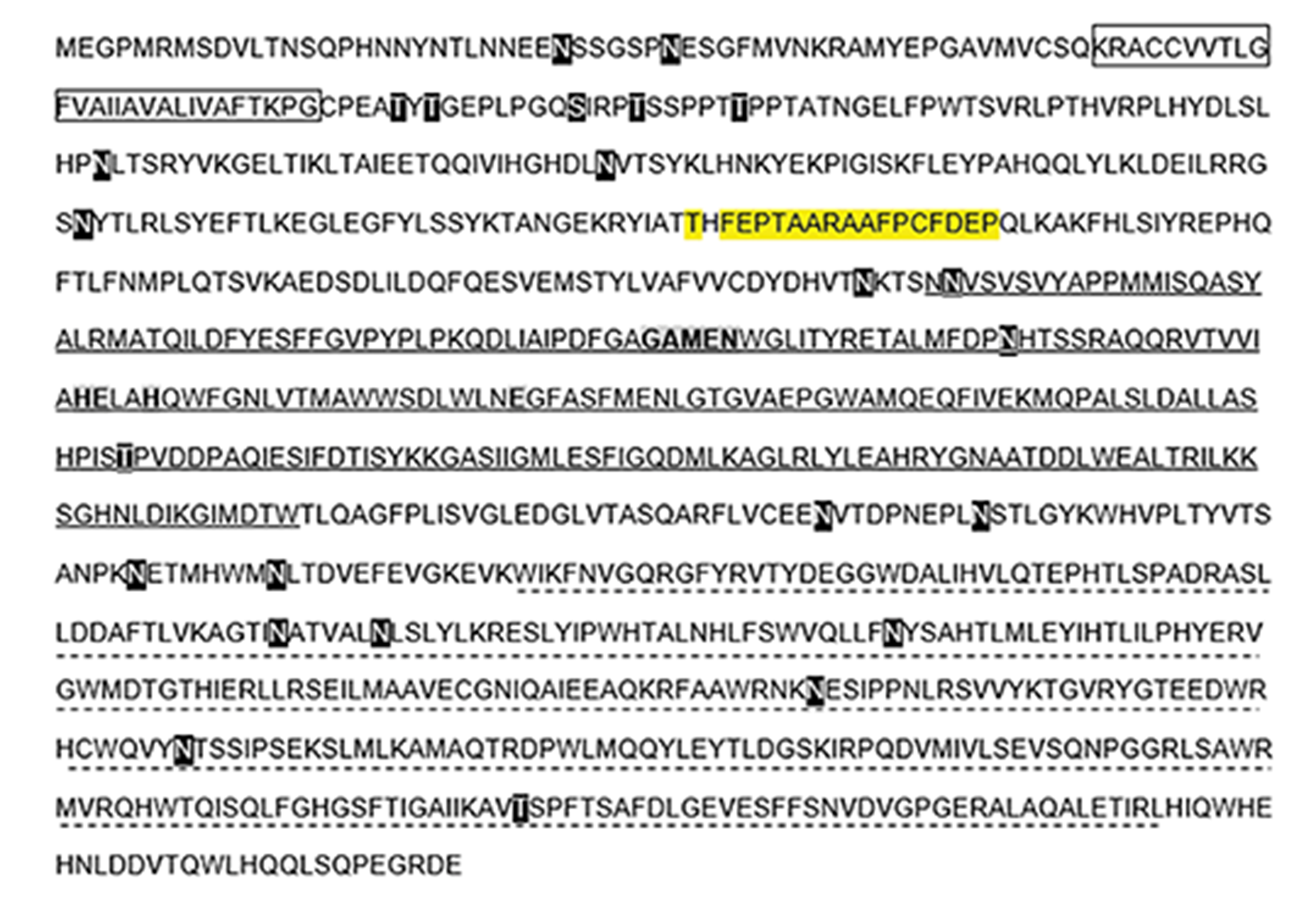

Supplement: S1 Fig — The putative N-terminal transmembrane domain is boxed. The GAMEN and HEXXH(X)18E zinc-binding site motifs are shown in bold with a grey background. The predicted Cry toxin binding region is highlight in yellow. Two conserved domains, the peptidase M1 domain and ERAP1-like C-terminal domain, are underlined and dash-underlined, respectively. Putative N-glycosylated asparagine residues predicted by the NetNGlyc 1.0 server, and putative O-glycosylated threonines and serine residues predicted by the NetOGlyc 3.1 server are shown in black. (TIF) [file ppat.1009463.s001.tif]

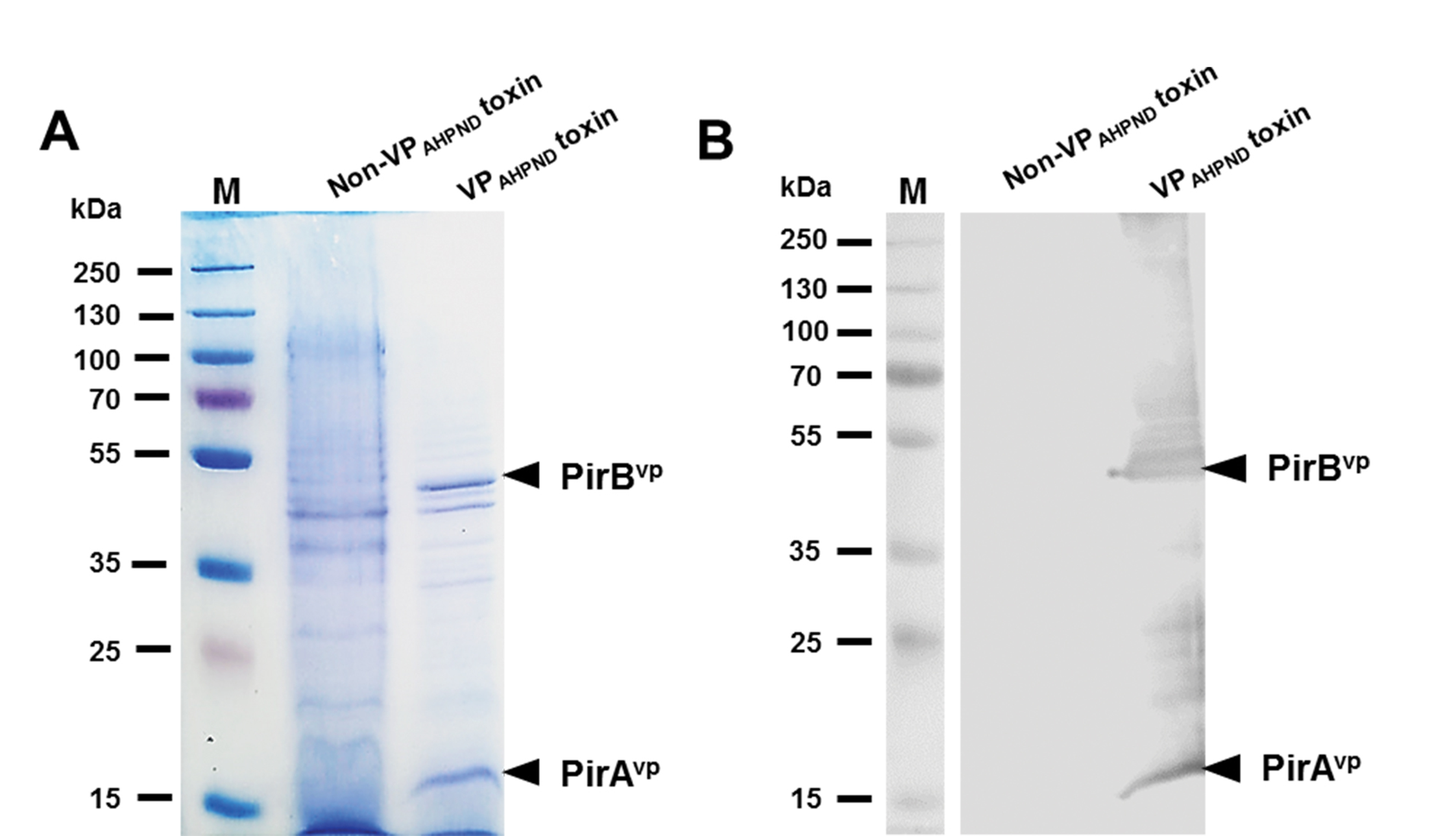

Supplement: S2 Fig — (A) SDS-PAGE analysis and (B) Western blot analysis with PirABvp polyclonal antibodies of the partially purified ammonium sulfate fractions from the culture medium of the non-AHPND isolate S02 and the VPAHPND isolate 5HP. The 2 major toxin bands (PirAvp at ~16 kDa and PirBvp at ~50 kDa) in lanes for partially purified 5HP proteins are absent from the proteins derived from S02. (TIF) [file ppat.1009463.s002.tif]

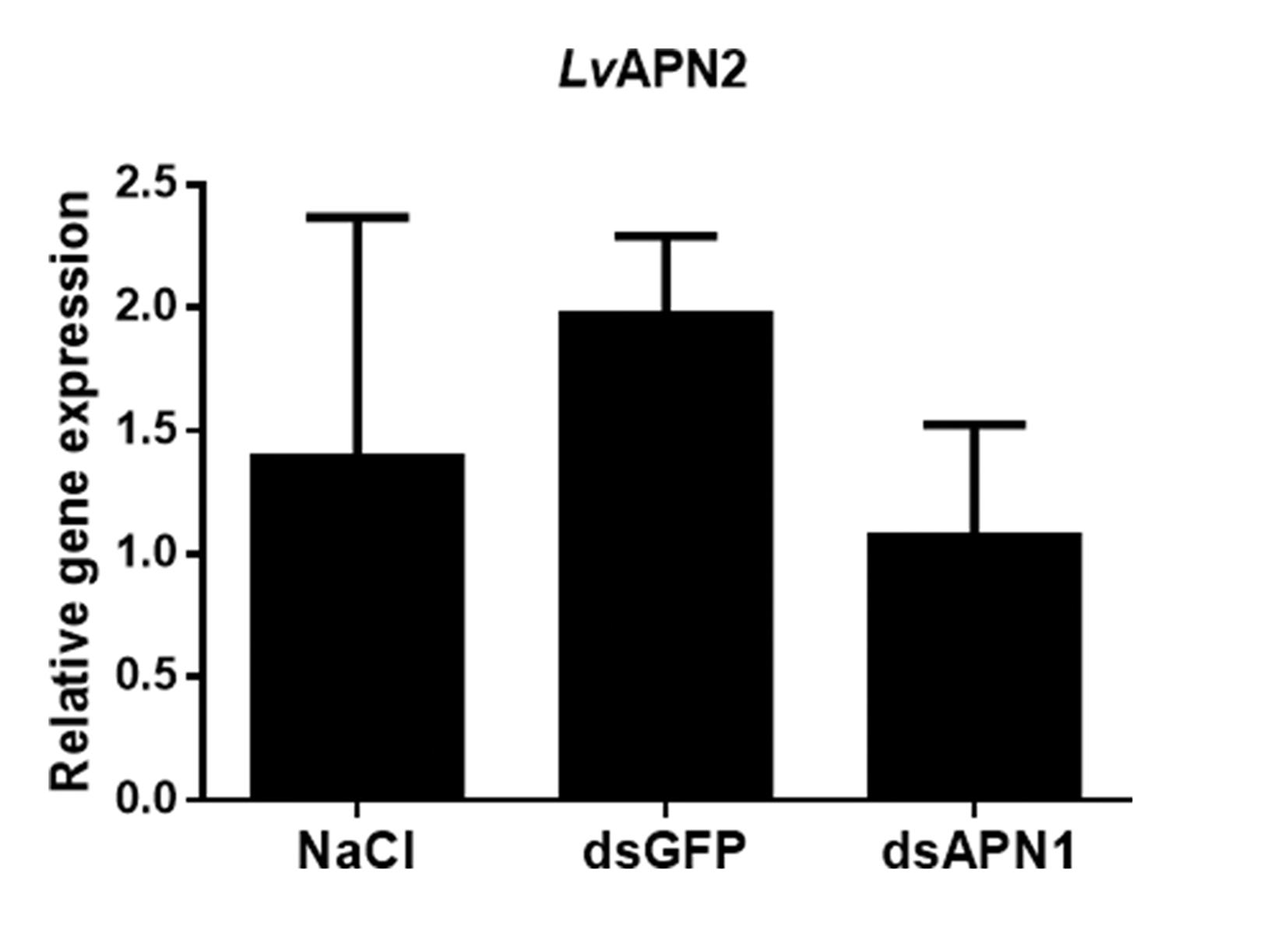

Supplement: S3 Fig — Shrimp were injected with 0.85% NaCl, 20 μg/g shrimp of dsGFP, or 20 μg/g shrimp of dsAPN1 were determined by qRT-PCR. Relative expression of LvAPN2 gene is shown here in relative to that of EF-1α. (TIF) [file ppat.1009463.s003.tif]

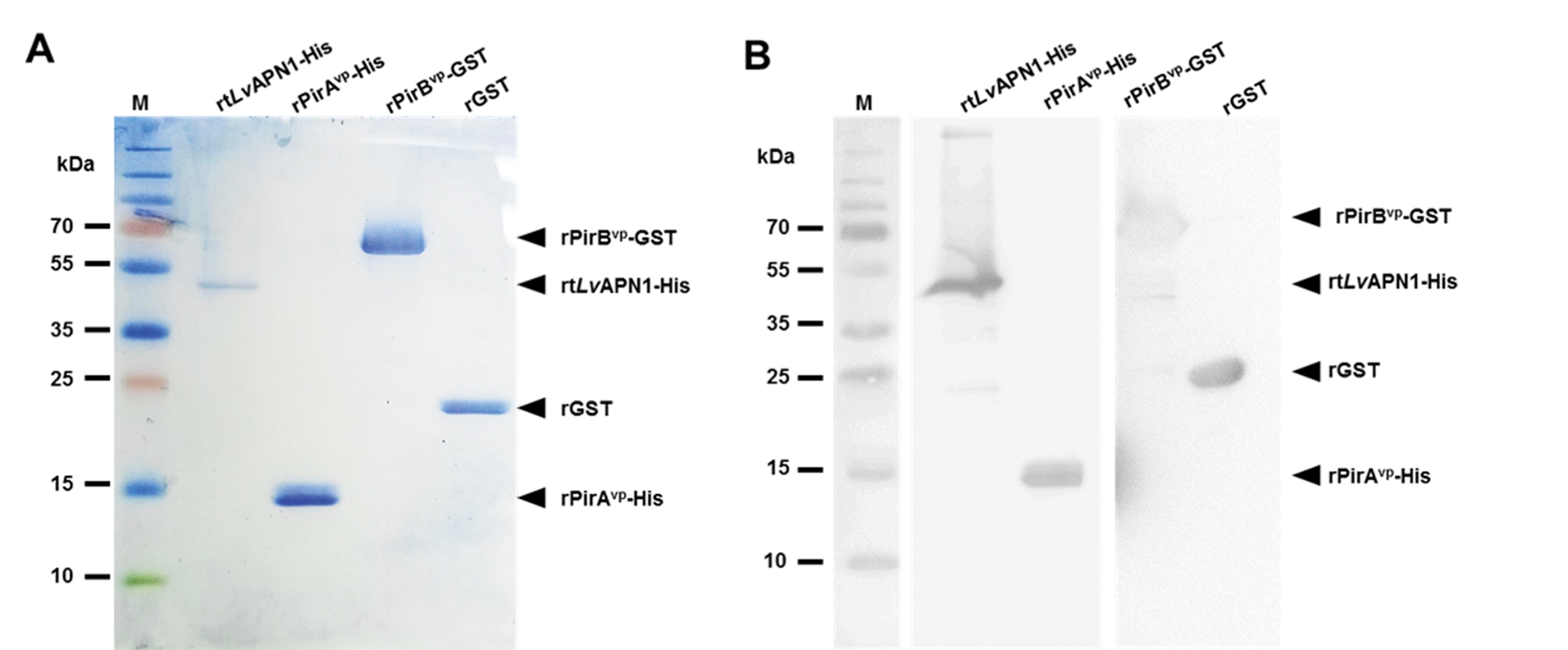

Supplement: S4 Fig — (A) SDS-PAGE analysis and (B) Western blot analysis with anti-His and anti-GST antibodies of recombinant truncated LvAPN1-His, rPirAvp-His, rPirBvp-GST and rGST protein overexpressed in E. coli. The His-tagged rLvAPN1 and rPirAvp were purified by Ni-NTA affinity chromatography. The deduced molecular weight for recombinant truncated LvAPN1-His and PirAvp-His were 53 and 16 kDa, respectively. The rPirBvp-GST fusion protein and rGST were purified by Sepharose 4B Glutathione beads. The estimated molecular weights for rPirBvp-GST and rGST were approximately 70 and 23 kDa, respectively. (TIF) [file ppat.1009463.s004.tif]
