# Supplementary material for: Cytotoxicity of Vibrio parahaemolyticus AHPND toxin on shrimp hemocytes, a newly identified target tissue, involves binding of toxin to aminopeptidase N1 receptor
Source: PLoS Pathog. 2021 Mar 26;17(3):e1009463. doi: 10.1371/journal.ppat.1009463 (PMC8041169; doi:10.1371/journal.ppat.1009463)
Supplement: Attachment — Submitted filename: Submit response to reviewers plos pathogen.docx [file ppat.1009463.s005.docx]

**Response to reviewers**

Dear Editor and reviewers,

Thank you for giving us the opportunity to submit a revised draft of our manuscript titled “Cytotoxicity of *Vibrio parahaemolyticus* AHPND toxin on shrimp hemocytes, a newly identified target tissue, involves binding of toxin to aminopeptidase N1 receptor” to PLOS Pathogens. We appreciate the time and effort that you and the reviewers have dedicated to providing your valuable feedback on our manuscript. We are grateful to the reviewers for their insightful comments on my paper. We have been incorporated changes to reflect the suggestions provided by the reviewers. We have highlighted and tracked the changes within the manuscript. Here is a point-by-point response to the reviewers’ comments and concerns.

**#Reviewer 1**

**Major Issues:**

While most of my comments are minor, it is important that the authors include data on how many replicates for each experiment. This was not consistently described. While not essential for the conclusions of the paper, it would definitely have been nice to see a binding curve for truncated rLvAPN1 with the heterodimer.

**Response:** We literally appreciate the suggestion. Sorry for not describing the number of replicates before. Now we have added the number of replicates for each experiment in the materials and methods section as suggested. (Fig. 1 Lines 354-355; 739; Fig.2: Lines 411-412; 747; Fig.3: Lines 440-441, 450; 754-755 ; Fig 4: Lines 461, 471-472, 495-496; 782-783; Fig 5: 510-511, 792)

For the second part of the comments, we agree that it is interesting to determine the binding assay for truncated r*Lv*APN1 with the heterodimer toxin; although, it is not essential for the conclusions of the paper. Previously, *in vitro* study by Lin et al., (2018) showed that the PirA^vp^/PirB^vp^ complex is unstable and has a low binding affinity. Therefore, we will further explore how this PirA^vp^/PirB^vp^ unstable complex interact with *Lv*APN1 and penetrate into shrimp cells.

**Reference**

Lin SJ, Chen YF, Hsu KC, et al. Structural Insights to the Heterotetrameric Interaction between the *Vibrio parahaemolyticus* PirA^vp^ and PirB^vp^ Toxins and Activation of the Cry-Like Pore-Forming Domain. Toxins. 11(4), 233 (2019).

**Minor Issues:**

1. Figures 1 and 2 are mis-ordered.

**Response:** Thank you for pointing out this error. The order of Figs. 1 and 2 has been updated.

2. Move Fig S4 to Fig S3 to stay consider with order of presentation in manuscript.

**Response:** Thank you for pointing out. Figs. S4 and S3 have been rearranged in order of presentation in manuscript. Changes have been made in the text (Lines173 and 236) and Figure legend.

3. Symbols in panel 3B almost completely illegible. Use color.

**Response:** Thank you for pointing out. The symbol style and line color of Fig. 3B have been changed as suggested. Figure 3 legend has been also updated (Lines 758-762).

4. Fig. 3C. Use of two different types of arrow heads would be clearer than the # and * symbols in pointing the eye to the relevant features.

**Response:** Thank you for pointing this out. We have changed the arrow styles used in Fig. 3C as suggested. The Fig. 3 legend has been updated (Lines 766-768).

5. Lines 233-234: “Assuming a one-site binding model, the apparent dissociation constants (Kd) of truncated rLvAPN1 to rPirA^vp^ and rPirB^vp^, as calculated from the saturation curves, were 3.157Å~10-6 M and 0. 499Å~10-6 M, respectively.” I think stating the apparent Kd as 3.2 µM and 0.5 µM would be more appropriate as ELISA’s are not high precision measurements.

**Response:** Thank you for your comment. The apparent *Kd* value has been expressed as 3.2 µM and 0.5 µM according to the reviewer’s suggestion (Lines 239-240).

6. The cartoon in Figure 7 might lead readers to think that only the first toxin requires a receptor. Once one is bound, the remain toxins bind by oligomerization. Assuming this is not the intended implication, I recommend removing the receptor for the toxin once the toxin is drawn in its oligomerized form. Authors can note that it is not clear if the receptor remains bound.

**Response:** We totally agree with your comment. The reviewer makes a great point. Our cartoon picture might mislead the reader that only the first toxin requires a receptor and the remaining toxins just form oligomer. As suggested, the receptor with the bound oligomerized toxin has been removed from Fig. 7.

7. Western blots using anti-His and anti-GST antibodies were used to confirm the expression of the respective proteins. Authors should state the source of the antibodies. If commercial, include the company or catalog number.

**Response:** Thank you for your comment. The anti-His and anti-GST antibodies are commercial products, so the company has been mentioned in Material and Method (Line 530), as suggested.

8. Fig 2c. How many replicates were performed?

**Response:** Thank you for your comment. Figure 2C (Now is Figure 1C in the revised manuscript) shows the tissue distribution analysis of *Lv*APN gene in shrimp. Results are representative of three biological replicates. We have added this information in Materials and Methods (Lines 354-355) and Figure 1 legend (Lines 739).

9. Fig. 1: How many replicates were performed?

**Response:** Thank you for your comment. In Fig. 1 (Now is Fig. 2 in the revised manuscript), all experiments were performed in triplicate. We have added this information in the Materials and Methods (Lines 411-412) and Figure 2 legend (Line 747).

10. Fig. 5: How many replicates were performed?

**Response:** Thank you for your comment. In Figure 5, all experiments were performed in triplicate. We have added this information in Materials and Methods (Lines 510-511) and Figure legend (Line 792).

**#Reviewer 2**

**Major Issues:**

1. Authors have not done paper review properly

1. The B Subunit of PirAB^vp^ Toxin Secreted from Vibrio parahaemolyticus Causing AHPND Is an Amino Sugar Specific Lectin.

2. PirAB^VP^ Toxin Binds to Epithelial Cells of the Digestive Tract and Produce Pathognomonic AHPND Lesions in Germ-Free Brine Shrimp. Especially the later paper demonstrate that PirAB^VP^ toxin are not specific to hepatopancreas but it can target and binds to midgut and hindgut region of digestive tract. So, I think these papers can be used for writing more interesting discussion.

**Response:** We thank the reviewer for this useful information. Literature search has been further performed and these references have been utilized where appropriate. According to Victorio-De Los Santos M et al. (2020), the property of PirB^VP^ toxin subunit as an amino sugar specific lectin supporting our finding that rPirB^VP^ binds to truncated rLvAPN1 with lower Kd than that of rPirA^VP^ has been discussed (Lines 309-313). In addition, the previous report by Kumar et al. (2019), suggested that the PirAB^VP^  binary toxins can binds to midgut and hindgut region of digestive tract causing cell damage. The introduction and discussion have been rewritten and this work has been cited (Lines 101-106 and Lines 279-281). Also, the reference section has been updated (Lines 666-669 and Lines 613-615).

**References:**

Victorio-De Los Santos M, Vibanco-Pérez N, Soto-Rodriguez S, Pereyra A, Zenteno E, Cano-Sánchez P. The B Subunit of PirAB^vp^ Toxin Secreted from *Vibrio parahaemolyticus* Causing AHPND Is an Amino Sugar Specific Lectin. Pathogens. 9(3),182 (2020).

Kumar V, Bels LD, Couck L, Baruah K, Bossier P, Broeck WVd. PirAB^VP^ Toxin Binds to Epithelial Cells of the Digestive Tract and Produce Pathognomonic AHPND Lesions in Germ-Free Brine Shrimp. Toxins. 11(12), 717 (2019).

**Minor Issues:**

1. Line 78- they are homologous to Photorhabdus luminescens insect-related (Pir) toxins PirA/PirB not PirAvp/PirBvp. Superscript Vp comes from Vibrio parahaemolyticus.

**Response:** Sorry for the typing error. As suggested by reviewer, “ *Photorhabdus luminescens* insect-related (Pir) toxins PirA^vp^/PirB^vp”^ has been changed to “*Photorhabdus luminescens* insect-related (Pir) toxins PirA/PirB” (Line 79).

2. Line 354 Why for bacterial challenge and toxin challenge assay different AHPND and non-AHPND strains were used?

**Response:** From our previous report, transcriptomic data are derived from shrimp infected with VP_AHPND_ TM strains and we reported that the VP_AHPND_ toxin partially purified from this strain can cause shrimp death upon challenge. However, the method used for determining the bacterial number of bacteria is not well established for TM strains but 5HP and S02 strains. Therefore, we used the VP_AHPND_ 5HP and non-VP_AHPND_ control S02 strains for bacterial challenge instead of TM and MC strains after we have confirmed the expression of *Lv*APN1 of shrimp challenged with VP_AHPND_ 5HP strains are correspondent to our transcriptomic data. So that, we can evaluate the number of AHPND virulence plasmids in the *Lv*APN1 silencing experiment. In case of toxin challenge, we found that partial purified VP_AHPND_ toxin from either 5HP or TM strains cause the same mortality rate of shrimp and same characteristics of AHPND. We thought that using protein extracted from either 5HP or TM strains does not change the conclusion of the study.

3. Line 349 If the strain is non-AHPND then how it will be VP_AHPND_ strain, (MC)?

**Response:** Sorry for the typing error. The mistake has been corrected as suggested. In the revised manuscript “ VP_AHPND_ strain MC” has been changed to “non-VP_AHPND_ strain MC” (Lines 365-366).
